# AI-Assisted Medical Documentation in a Multilingual Swiss Health Care System: Proof-of-Concept Study

**DOI:** 10.2196/77351

**Published:** 2026-06-05

**Authors:** Mateusz Gładysz, Fabrizio Fiumedinisi, Felice Burn, Nikki Rommers, Pietro Giovanoli, Jan Alexander Plock

**Affiliations:** 1 Department of Plastic Surgery and Hand Surgery, Kantonsspital Aarau, Tellstrasse 25, Aarau, 5001, Switzerland; 2 AI & Data Science CoE, Kantonsspital Aarau, Aarau, Switzerland; 3 University of Basel, Basel, Basel-City, Switzerland; 4 Department of Plastic Surgery and Hand Surgery, University Hospital of Zurich, Zurich, Switzerland

**Keywords:** artificial intelligence, natural language processing, speech recognition software, electronic health records, documentation, multilingualism, efficiency, organizational, burnout, professional, Switzerland, generative AI scribes, ambient clinical documentation, comparative study

## Abstract

**Background:**

Medical documentation imposes a significant administrative burden on physicians and reduces time for direct patient care. Artificial intelligence (AI)-assisted tools such as automatic speech recognition and large language models (LLMs) promise to reduce this burden, but their performance in multilingual environments has not been explored. Switzerland is highly multilingual, and non-native German-speaking physicians may find documentation particularly challenging.

**Objective:**

This study aimed to compare the efficiency and documentation quality of four clinical documentation workflows—including both AI-assisted and traditional methods—in a Swiss tertiary hospital setting characterized by linguistic diversity.

**Methods:**

In this proof-of-concept study at a Swiss tertiary hospital (Department of Plastic and Hand Surgery, Cantonal Hospital Aarau), two physicians—a native Swiss German speaker and a non-native German speaker—documented encounters with simulated patients having common hand disorders. Four documentation workflows were tested: (1) traditional dictation with transcription by a secretary; (2) real-time dictation using speech recognition software for voice to text transcription; (3) postencounter dictation transcribed by an AI (Whisper) and processed by a GPT-based agent; and (4) AI-assisted ambient dictation of entire appointments using audio recording and automatic transcription. Documentation efficiency was measured by recorded physician time, and note quality was assessed using a modified Physician Documentation Quality Instrument (PDQI-9) scored by three different LLMs. To protect patient privacy, only synthetic (simulated) patient data were used.

**Results:**

AI-assisted workflows—particularly workflow 4 (AI-assisted ambient dictation)—produced the shortest physician documentation times per report. In post-hoc comparisons, workflow 4 was significantly faster than solely the speech recognition software workflow (workflow 2) for both physicians (adjusted *P*<.001). For the non-native speaker, workflow 4 was not significantly faster than traditional dictation (workflow 1) after adjustment (*P*=.08). LLM evaluators assigned high absolute scores (median PDQI-9 >47/50); however, inter-rater reliability was poor (Krippendorff’s alpha=−.433, 95% CI: −0.444 to −0.416), indicating systematic disagreement that precludes definitive conclusions about documentation quality from these scores alone.

**Conclusions:**

AI-assisted documentation demonstrated significant time savings for the native speaker, though the reduction for the non-native speaker did not reach statistical significance in this pilot (*P*=.08). Such tools show potential to alleviate the linguistic challenges faced by non-native speakers, reduce administrative burdens, and enable physicians to spend more time with patients. However, the inconsistency of AI-based quality scoring suggests that LLMs cannot yet reliably replace human evaluation. Future studies should evaluate these workflows in real-world clinical implementation, address data privacy and security issues, and include human evaluators to validate the benefits observed in this study.

## Introduction

Medical documentation is essential for health care delivery but imposes a substantial administrative burden on physicians. Studies indicate that growing documentation requirements greatly reduce time available for direct patient care, with estimates that physicians spend nearly twice as much time on administrative tasks as on patient interaction [1]. This imbalance contributes to physician burnout—a rising concern in health care systems worldwide, including in Switzerland [2]. Burnout adversely affects physicians’ well-being and has been linked to compromised patient safety, lower care quality, and reduced health care efficiency.

Switzerland’s linguistic diversity creates unique challenges for health care communication and documentation. The country has four national languages (ie, German, French, Italian, and Romansh) and numerous regional dialects [3]. In German-speaking regions, physicians and patients often switch between Swiss German dialects and standard German, complicating both verbal communication and written notes. English is also commonly used as a lingua franca in medical settings [4]. Non-native speakers comprise a significant portion of the Swiss medical workforce—over 40% of physicians practicing in Switzerland received their medical education abroad [5], —and they face added difficulties documenting and communicating efficiently across multiple languages and dialects. Patients further contribute to this linguistic complexity by speaking various dialects or languages, adding another layer of difficulty to clinical interactions. For example, patients in German-speaking areas might use local Swiss German dialects, standard German, or even Italian or French. The multiplicity of languages and dialects makes accurate, efficient documentation challenging, particularly when using speech recognition technologies not optimized for regional dialects or code-switching. These linguistic factors can increase documentation time, cause misunderstandings, and lead to variability in the quality of medical records.

Advancements in artificial intelligence (AI)—particularly in natural language processing and speech recognition—offer promising solutions to ease the documentation burden [6] Recent competitive analyses show that commercial AI scribes can generate notes rapidly (≈1 min for standardized 15-min encounters) but performance and error profiles vary between products, warranting ongoing evaluation [7]. Tools such as large language models (LLMs) and speech-to-text systems can generate high-quality medical notes and reduce documentation time, and some are already in commercial use [8] These AI-assisted tools can transcribe spoken language, interpret clinical context, and produce structured notes that integrate with electronic health record systems. However, most studies of such technologies have been conducted in monolingual, predominantly English-speaking environments. A significant gap in the literature remains regarding their effectiveness in multilingual, linguistically complex settings like Switzerland, where the mix of languages and dialects poses additional challenges for AI models trained primarily on standard language data.

Moreover, implementing AI-assisted documentation tools raises important ethical and practical considerations [9]. Data privacy and security are paramount, especially when sensitive patient information is processed via cloud-based AI services [10]. Compliance with regulations such as the European Union’s General Data Protection Regulation (GDPR) is essential to protect patient confidentiality [11]. Additionally, AI models may exhibit biases or performance limitations when handling languages or dialects that were not well represented in their training data [12]. These concerns must be addressed to ensure the safe and effective integration of AI technologies into clinical practice.

This study aims to evaluate the efficiency and quality of AI-assisted medical documentation workflows in a linguistically diverse Swiss tertiary hospital setting. By comparing traditional and AI-assisted documentation methods used by both native and non-native German-speaking physicians, we seek to understand how these technologies perform in a real-world multilingual clinical environment. The findings are intended to inform the implementation of AI documentation tools in health care systems facing similar linguistic challenges, ultimately contributing to improved physician efficiency and patient care.

## Methods

### Study Design

We conducted a proof-of-concept observational study at the Department of Plastic and Hand Surgery, Cantonal Hospital Aarau AG (Aarau, Switzerland). The primary aim was to compare the efficiency and documentation quality of four medical documentation workflows (traditional vs AI-assisted) in a controlled, simulated clinical setting involving common hand disorders. This study design enabled a standardized comparison of workflows while adhering to ethical requirements for patient privacy and data protection.

### Participants and Generation of Simulated Encounters

The study involved two physician participants: one native Swiss German–speaking consultant in hand surgery and one non-native German–speaking consultant in plastic surgery. Both physicians were experienced clinicians in the department with equivalent professional experience. We conducted twelve simulated patient encounters featuring common hand disorders such as trigger finger, Dupuytren contracture, de Quervain tenosynovitis, and carpal tunnel syndrome. Clinical scenarios and referral letters were initially generated using ChatGPT (GPT-4), then carefully reviewed for clinical accuracy by the first author.

Simulated patients were portrayed by residents and interns familiar with the medical conditions and daily hand surgery practice. Each consultant completed encounters with six simulated cases, ensuring balanced linguistic diversity per consultant as follows:

- Standard German speakers (n=2): actors who spoke standard German as their first language.
- Non-native German speakers (n=2): actors who spoke German as a second language (first language Italian), representing individuals from Ticino or Italy.
- Swiss German dialect speakers (n=2): actors who spoke different Swiss German dialects (eg, from Aargau and Bern).

Some actors portrayed scenarios for both consultants, although not all did. Specific actor-case assignments were not systematically recorded.

This diversity in linguistic backgrounds was designated to simulate realistic clinical interactions, ensuring each documentation workflow was tested under representative conditions reflective of Switzerland’s multilingual health care environment.

### Workflows Tested

We compared four documentation workflows, each with a different level of technological assistance :

Workflow 1: Traditional Dictation to a Secretary – After each simulated patient encounter, the physician dictated the clinical notes, which were transcribed by a medical secretary. The physician later reviewed and corrected the transcribed note in the electronic health record (EHR) system.

Workflow 2: Speech Recognition Software – The physician used Dragon Medical One® speech recognition software to dictate notes directly into the EHR during or immediately after each encounter, correcting errors in real time without a secretary.

Workflow 3: AI Transcription + GPT Agent – After each encounter, the physician dictated the notes, which were transcribed using OpenAI Whisper (Large v3) speech-to-text. A custom GPT-based agent then processed the transcript to generate a structured draft of the note. The physician reviewed and edited the AI-generated note before finalizing it in the EHR. Whisper Large v3 was used out of the box, with no fine-tuning, vocabulary customization, or site-specific optimization.

Workflow 4: Ambient Dictation – The entire patient–physician encounter was audio-recorded (using a smartphone app). After the appointment, the physician added any examination details by dictating into the recorded audio. The recording was transcribed using the open-source Whisper Large v3 model, and the transcript was then processed by a custom GPT-based system (MediBrief Creator©) to produce a draft note. The physician reviewed the draft for accuracy and added any corrections before integrating the note into the EHR. Whisper Large v3 was used out of the box, with no fine-tuning, vocabulary customization, or site-specific optimization.

#### System Instructions for “MediBrief Creator”

##### Role

Create “Arztbriefe” (medical reports) in German from transcripts.

Structure:

- *Hauptdiagnose/Nebendiagnose:* Diagnosis with ICD-10 code
- *Anamnese:* Subjective history (complete sentences, past tense/subjunctive)
- *Befund:* Objective findings (complete sentences, no bullet points)
- *Beurteilung/Procedere:* Summary and plan

##### Key Guidelines

Use formal terminology; do not fabricate information; preserve uncertainty if transcript is unclear; “as much as necessary, as little as possible.”

### LLM Pipeline (Reproducibility)

Audio was transcribed with Whisper Large v3 and then summarized by a fixed, GPT 4o -based agent (“MediBrief Creator” [13]), using a stable prompt stack: (1) section templating (History, Exam, Assessment, Plan); (2) conservative synthesis (“preserve uncertainty; do not invent”); (3) problem-oriented summary with code suggestions; (4) style normalization; and (5) self-flagging for low-confidence segments. The same prompts/parameters were used for all cases; no mid-study prompt editing occurred (Table 1).

**Table 1.** Key characteristics of each workflow (physician’s role, technology used, and notable features).

Attribute	Workflow 1: Traditional dictation to secretary	Workflow 2: Dragon Medical One software	Workflow 3: Dictation with Whisper V3 and GPT Agent	Workflow 4: Ambient dictation with full appointment recording and GPT processing
Description	Physicians dictate notes post-encounter; secretaries transcribe and return for review.	Physicians use speech recognition software to dictate directly into EHR^a^, correcting errors in real-time.	Physicians dictate notes; AI^b^ transcribes and generates structured notes for review.	Entire patient encounters are recorded; AI transcribes and generates notes; physicians add missing details post-encounter.
Physician's role	Dictate patient notes Review and correct transcribed notes	Dictate notes using Dragon Medical One Edit and correct transcribed text within the EHR	Dictate patient notes Review and correct AI-generated notes	Conduct patient encounters Provide additional clinical details post-encounter Review and correct AI-generated notes
Technology used	Digital voice recorder Human transcription by secretary	Dragon Medical One software EHR system	OpenAI Whisper V3 (speech-to-text) Custom GPT agent (note generation)	Smartphone recording app OpenAI Whisper V3 Custom GPT agent
Key features	Established practice Division of labor Potential delays due to transcription time	Real-time transcription Immediate self-correction No secretary involvement	AI-assisted transcription and note generation Structured notes with minimal formatting effort Inclusion of *ICD-10* codes by AI	Passive recording of encounters AI-generated notes from full transcripts Minimal active documentation during encounters Inclusion of *ICD-10* codes by AI
Rationale	Serves as control workflow Reflects standard practice	Tests efficiency of speech recognition technology without AI language models	Evaluates combined use of advanced speech-to-text and AI language models Aims to reduce documentation time and effort	Tests the concept of an ambient AI scribe Aims to further reduce active documentation time

a EHR: electronic health record.

b AI: artificial intelligence.

### Efficiency Measurement

An independent observer measured the time each physician spent on documentation tasks in each workflow using a stopwatch. For Workflow 2, 'dictation’ was defined as the time the microphone was active, while ’correction’ was defined as time spent on manual input (typing/mouse); we acknowledge these often overlap in practice. Background processing time (eg, AI transcription or note generation by the GPT agent) was not counted, under the assumption that those processes occur automatically without the physician’s active involvement. This measurement method isolated the physician’s active documentation time for each workflow.

### Quality Assessment

Note quality was scored using a modified PDQI-9 [8,14] approach previously used for AI-scribe evaluation. We rated 10 criteria on 5-point Likert scales (total 0‐50): Accuracy, Thoroughness, Usefulness, Organization, Comprehensibility, Succinctness, Synthesis, Internal Consistency, Lack of Hallucination, Lack of Bias. No item weighting was used. We reused the published “modified PDQI-9” approach and list the full criteria here to ensure reproducibility without an appendix.

### LLM Raters

Three independent LLMs (Claude 3 [15], OpenAI o1-preview [16], and GPT-4 [17]) rated all notes. Inter-rater reliability was analyzed separately in the Statistical Analysis section using Krippendorff’s α.

### Quality Assessment Prompt Instructions

The quality assessment used a two-tier approach (Textbox 1). For the attributes “Free from Hallucination” and “Free from Bias” workflow 2 was designated as the reference standard because it represents physician-authored, physician-corrected documentation — the physician dictated into Dragon Medical One, reviewed the output in real time, and manually corrected errors before finalizing the note. Notes from Workflows 1, 3, and 4 were scored by comparison against the corresponding Workflow 2 note for the same patient. For these two attributes, Workflow 2 notes automatically received a score of 5/5. For the remaining eight PDQI-9 attributes (Accuracy, Thoroughness, Usefulness, Organization, Comprehensibility, Succinctness, Synthesis, Internal Consistency), each note was evaluated independently without an external reference standard. The LLMs’ assessment of these attributes therefore reflects perceived internal plausibility and linguistic quality rather than verification against clinical ground truth.

Textbox 1. **Quality assessment instructions.**

“In this project acts as a professional medical documentation reviewer, assessing medical notes based on the Modified Physician Documentation Quality Instrument (PDQI-9). It focuses on the following sections: Diagnose, Nebendiagnose, Anamnese, Befund, and Beurteilung. You will not provide medical advice or diagnoses. You will strictly focus on assessing the quality of the documentation.

**Assessment Attributes:**

You will rate the notes on the following ten attributes:

1. Accurate
2. Thorough
3. Useful
4. Organized
5. Comprehensible
6. Succinct
7. Synthesized
8. Internally Consistent
9. Free from Hallucination
10. Free from Bias

**Scoring System:**

Each attribute will be scored on a scale from 1 to 5 after in-depth analysis of the note.

- Score 1: Terrible, the note does not fulfill this attribute.
- Score 5: Excellent, the note fully meets this attribute with a perfect score.

**Workflow-Specific Scoring:**

- For notes generated in Workflow 2 the attributes **Free from Hallucination** and **Free from Bias** will automatically receive a score of 5/5.
- For notes generated in Workflow 1, 3 and Workflow 4, these attributes will be assessed through content comparison to the notes from Workflow 2 for the same patient.

**Tone and Interaction:**

The tone will remain professional, maintaining a formal and respectful interaction.

**Use Case:**

This project is designed for use in a research to assess 48 medical notes created across 4 workflows for 12 test patients.

**Output Format:**

Results will be presented in a table format suitable for statistical analysis, with an additional summary score for each assessed note.”

Note: The above instructions are reproduced verbatim as deployed. They contain grammatical errors (eg, missing subject in “In this project acts as.“; “for use in a research” instead of “for use in research”) because they were originally authored as system instructions for an OpenAI Custom GPT platform, where the agent subject is implicit. These errors are acknowledged as a limitation; prompt phrasing can influence LLM behavior, and the grammatical imprecision may have introduced additional variance into the scoring process.

### Ethical Considerations

The study used only simulated patient data to avoid privacy concerns and comply with data protection regulations (eg, GDPR) [9]. We evaluated the AI tools (including cloud-based transcription and note-generation services) in a simulated environment to ensure feasibility while upholding data privacy standards.

### Statistical Analysis

The main outcome of the study is efficacy of the workflows, expressed in the total time the physician needs to invest in the report standardized by the consultation time. This standardized total time consists of dictation and correction time. The efficacy was compared between workflows and native language of the physician. Given the small sample, nonparametric statistical models were used. We use the so-called F1_LD_F1 model described by Brunner & Langer [18] for the outcome time the physician spends on the report. This test is a nonparametric test with one within-patient factor (ie, workflow), and one between-patient factor (ie, physician language). The test statistic used is the ANOVA-type statistic (ATS), which provides asymptotically valid *P* values without relying on parametric assumptions, and would correspond to a parametric mixed-effects ANOVA. We are interested in the interaction effect of workflow and physician language on the time it takes to produce the report, as well as the two main effects of workflow and physician language. This method is implemented in the R package *nparLD2* [19]. We report the unadjusted *P* values for the interaction effect and main effects. In case of a significant interaction effect, we continued with pairwise comparisons of the times for the workflows using Dunn’s test of multiple comparisons with Bonferroni correction to control for the family-wise error rate, and report the rank-biserial correlation *rr*r as an effect size for each pairwise comparison. We report the adjusted *P* values of the multiple comparisons. This analysis was repeated for the dictation and correction time separately.

For the secretary time, which was only available for one workflow, we compared native and non-native speakers using the Wilcoxon rank-sum test.

Inter-rater reliability among the three LLMs was quantified using Krippendorff’s α, calculated on the total score using an interval-scale metric, which accounts for the magnitude of differences between ratings. The coefficient ranges from –1 (perfect systematic disagreement) to 1 (perfect agreement), with 0 indicating agreement no better than chance. Confidence intervals were derived via bootstrap resampling with 1000 iterations.

## Results

### Time Efficiency

**Figure 1.**
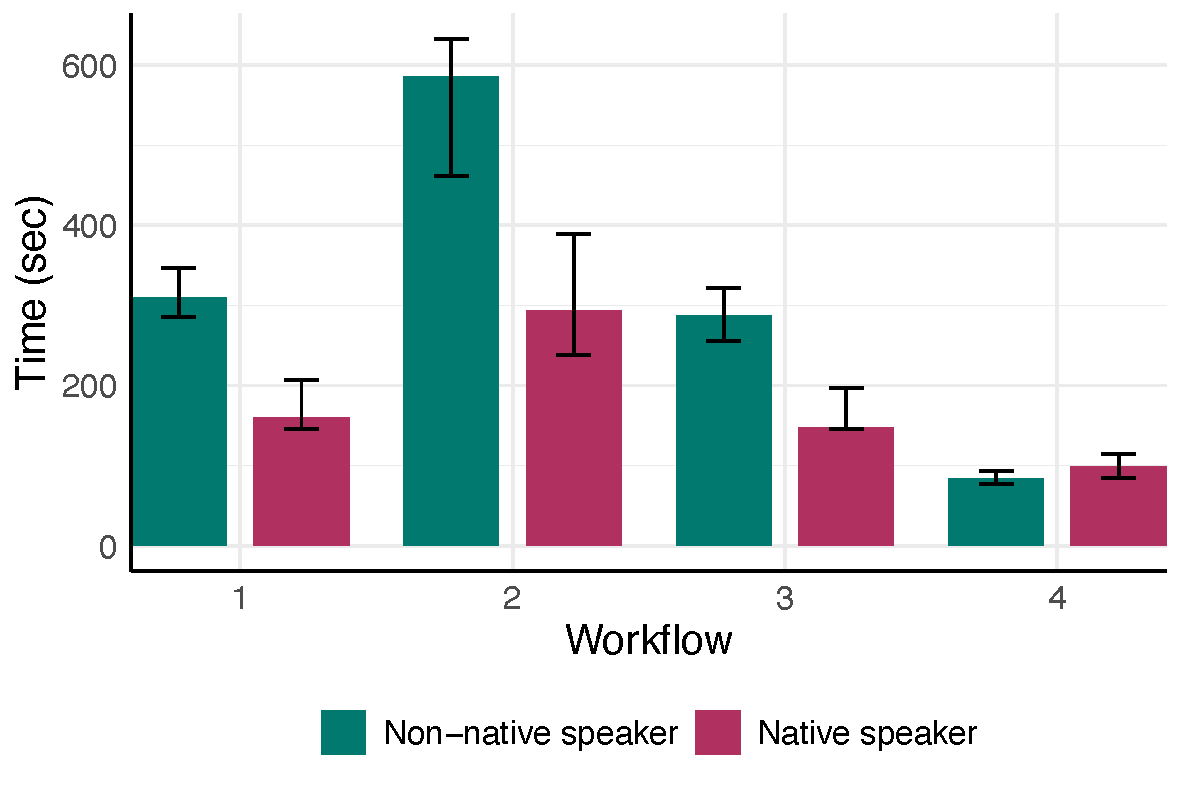
Physician’s median time (in seconds) to finalize the report stratified by workflow and native language. Bars show the interquartile range (25th to 75th percentile), and different colors indicate workflow–language combinations.

We assessed the documentation time outcomes for each workflow in each physician group (native vs non-native German speaker) (Figures1  2, Table 2).

**Figure 2.**
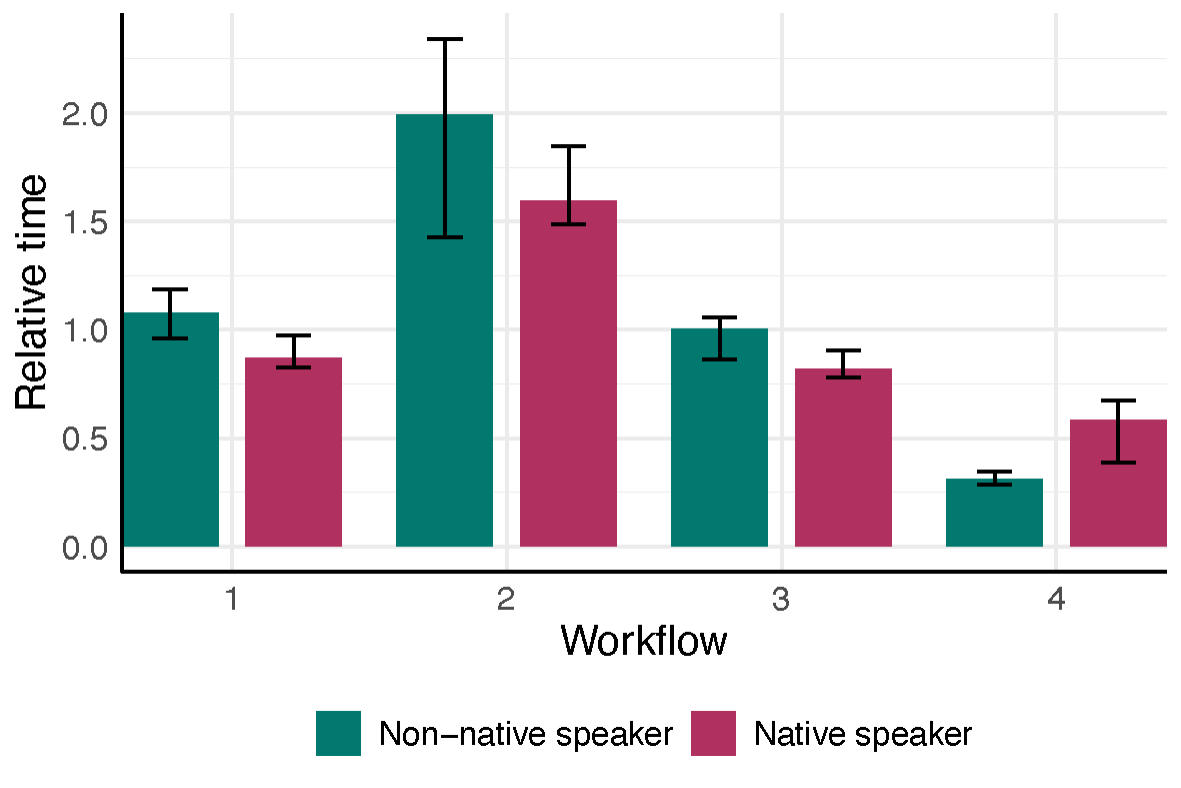
Physician’s median relative time to finalize the report (absolute time needed (sec) divided by the consultation time (sec)) stratified by workflow and the physician’s native language. Bars show the interquartile range (25th to 75th percentile), and different colors indicate workflow–language combinations.

**Table 2.** Median and interquartile range [25% percentile, 75% percentile] of total documentation time for each workflow by physician. The median [Q1, Q3] physician times (seconds) required to finalize a note.

Workflow	Native speaker	Non-native speaker
Workflow 1: Traditional Dictation	160 [146, 207]	310 [285, 347]
Workflow 2: Dragon Medical One Software	294 [238, 389]	586 [462, 632]
Workflow 3: Whisper V3 and GPT Agent	148 [145, 197]	287 [256, 321]
Workflow 4: AI-Assisted Ambient Dictation	99 [85, 114]	84 [77, 93]

**Figure 3.**
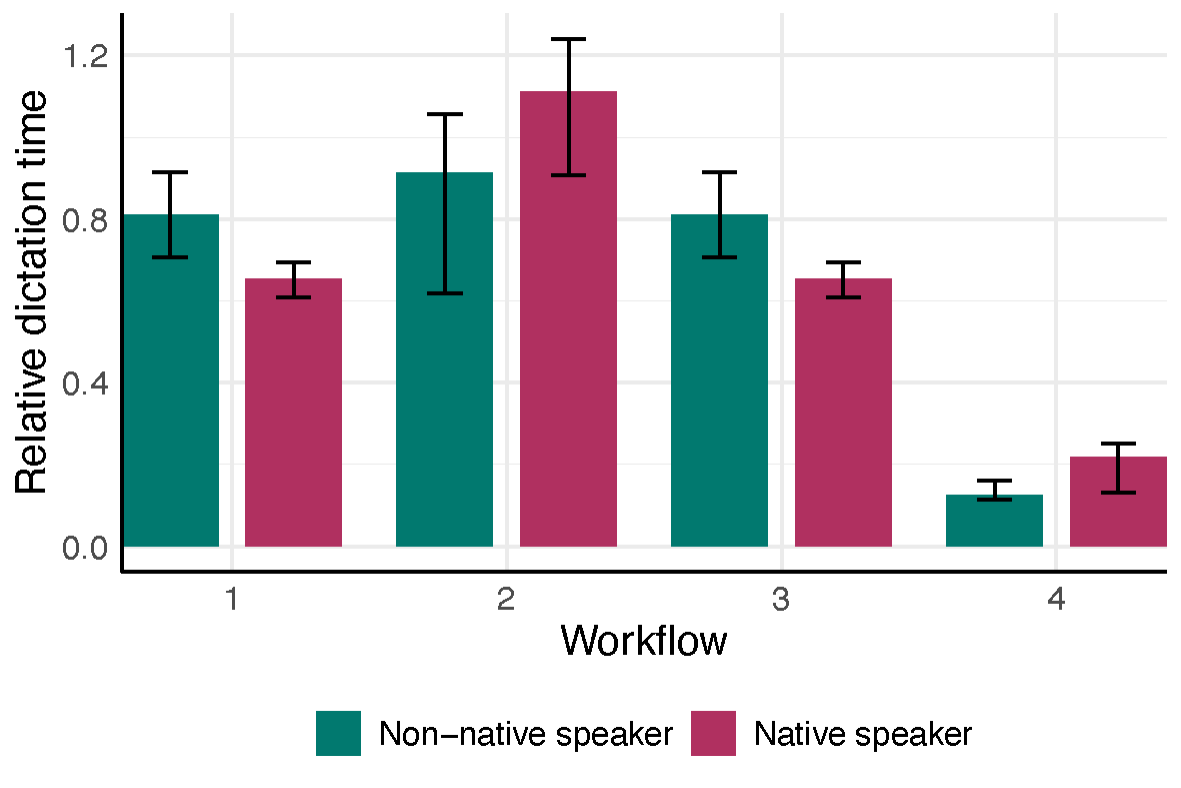
Physician’s median relative dictation time (absolute dictation time (sec) divided by the consultation time (sec)) stratified by workflow and the physician’s native language. Bars show the interquartile range (25th to 75th percentile), and different colors indicate workflow–language combinations.

**Figure 4.**
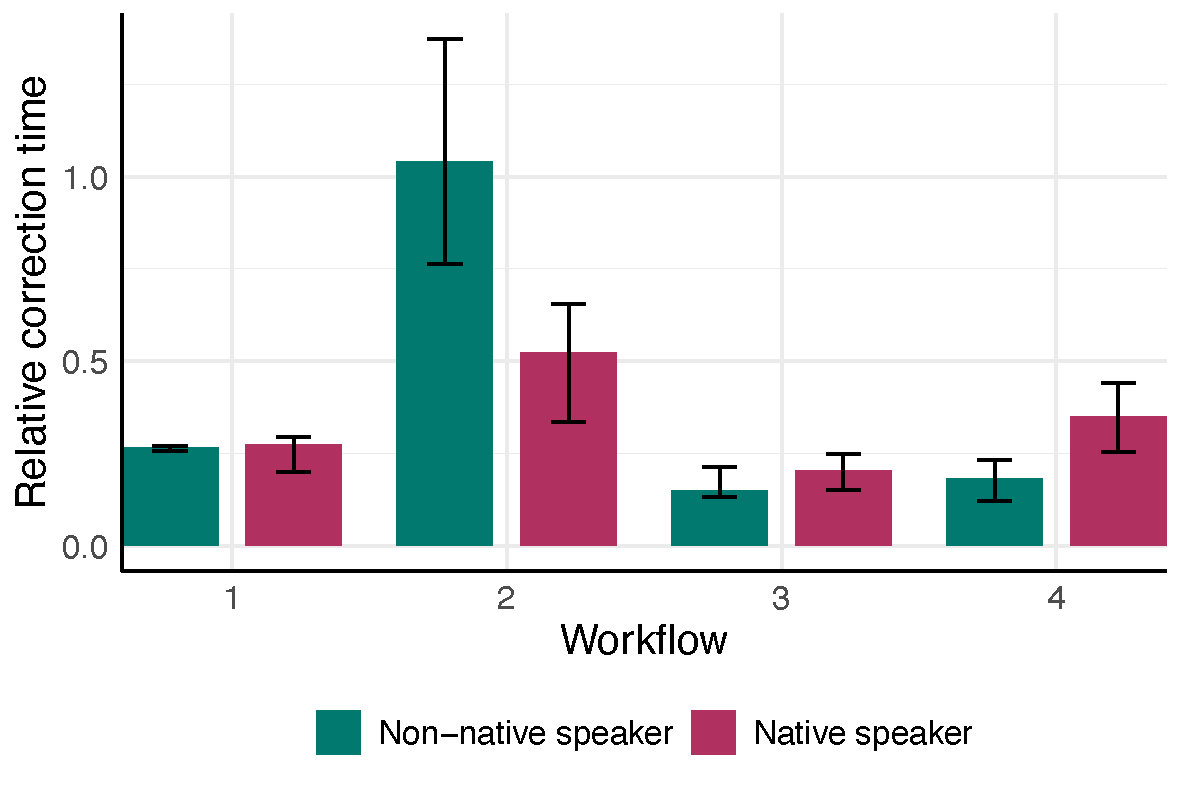
Physician’s median relative editing/correction time (absolute editing/correction time (sec) divided by the consultation time (sec)) stratified by workflow and the physician’s native language. Bars show the interquartile range (25th to 75th percentile), and different colors indicate workflow–language combinations. In Workflow 1, physician correction time was minimal but non-zero (median 76s for non-native; 45.5s for native).

Across the two physicians, the nonparametric ANOVA indicated a significant interaction between workflow and physician language (*P*=.001) and a strong main effect of workflow on documentation time (*P*<.001), with no significant main effect of physician language (*P*=.44) (Figure 3). In post-hoc comparisons for the native German-speaking physician, workflow 4 (ambient AI dictation) was significantly faster than workflow 2 (speech recognition software; adjusted *P*<.001), while no other pairwise differences were significant. For the non-native speaker, workflow 4 was also faster than workflow 2 (adjusted *P*<.001) but did not differ significantly from workflow 1 (traditional dictation; adjusted *P*=.08). Workflow 4 could save 62.9% of time (IQR 56.25, 77.88) for a native speaker, compared to a saving of 83.65% (IQR 80.52, 88.65) for the non-native speaker (Figure 4). No other between-workflow differences reached significance for the non-native speaker.

### Practical time savings

Using workflow medians, WF4 reduced active documentation time by ≈3.3 minutes versus WF2 for the native physician (294s vs 99s) and by ≈8.4 minutes for the non-native physician (586s vs 84s).

### Documentation Quality

LLM evaluators assigned high absolute PDQI-9 scores across all workflows (overall mean 46.7/50, SD 0.4); however, these scores must be interpreted with caution given the poor inter-rater reliability reported below. Notably, for the eight attributes assessed without an external reference standard — including “Accuracy” and “Thoroughness” — the LLM scores represent perceived plausibility and internal coherence rather than verified factual correctness, as no ground-truth transcript of the simulated encounters was available to the evaluators. Workflow 4 had the highest mean quality score (47.3, SD 1.0), followed by Workflow 1 (46.7, SD 1.2), Workflow 2 (46.3, SD 1.5), and Workflow 3 (46.3, SD 1.5). The largest absolute difference in mean scores between any two workflows was approximately 1 point.

### Quality Criteria Breakdown

**Figure 5.**
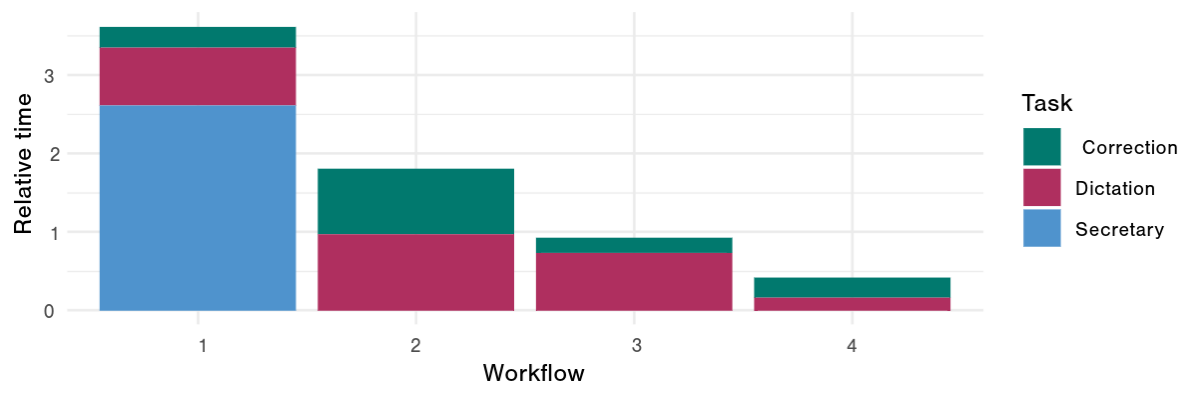
Decomposition of the relative time the of the different workflows into components dictation time, correction time, and secretary time. Secretary time reflects a nonphysician resource; it represents the time that the secretary needs to type and format the medical note from the dictated transcript; it is visualized only to contextualize task shifting across workflows and is not counted toward the primary physician-time outcome.

On each PDQI-9 quality criterion, all workflows performed similarly. For example, *Accuracy* was rated 5.0 (out of 5) for Workflows 1, 2, and 4, and 4.7 for Workflow 3. *Thoroughness* was scored 4.3 for Workflows 1‐3 and 4.7 for Workflow 4. Other criteria such as *Usefulness*, *Organization*, *Synthesis, Internal Consistency*, *Lack of Hallucination*, and *Lack of Bias* all had mean scores between 4.7 and 5.0 for every workflow. The *Comprehensibility* score was 4.5 for all workflows, and *Succinctness* was 4.0 for all. Criterion-level scores were similar across workflows, though the reliability of these scores is limited by the systematic disagreement among LLM evaluators (see Figure 5 for a radar chart of criteria scores).

**Figure 6.**
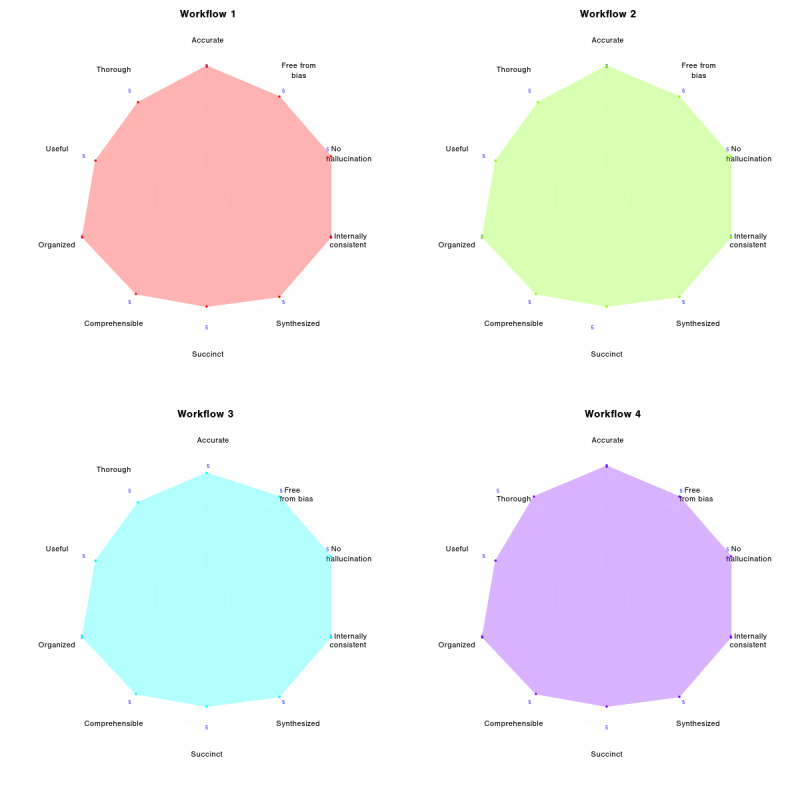
Radar chart of documentation quality criteria (PDQI-9,Physician Documentation Quality Instrument (9-item adaptation) ) scores for each workflow.

Detailed scores for each criterion are presented in radar plots 1‐4 (Figure 6).

### LLM Scoring Details

The three AI evaluators (LLMs) showed broadly consistent scoring patterns across workflows. *Anthropic Claude 3* assigned total quality scores ranging from 45 to 48 out of 50, with Workflow 4 receiving the highest score among those. *OpenAI o1-preview* tended to give slightly higher scores overall—Workflows 3 and 4 each attained a perfect total score of 50 with this model. *GPT-4* also scored all workflows highly, though it gave Workflows 3 and 4 slightly lower total scores (44) than Workflows 1 and 2 (46). Despite minor model-to-model variations, all LLMs confirmed that Workflow 4’s notes were of quality comparable to or slightly better than those of the other workflows, reinforcing the finding of no major quality degradation with AI assistance. Agreement between the three LLMs on the total score (interval scale) was assessed using Krippendorff’s α. The coefficient was –0.433 (95% CI −0.444 to −0.416), indicating systematic disagreement beyond what would be expected by chance. This negative value reflects that the LLMs not only failed to agree, but tended to assign divergent scores to the same cases.

### Summary of Results

In summary, *Workflow 4* (ambient AI dictation) had the shortest documentation time overall with LLM-assigned quality scores comparable to the other workflows, though the reliability of these scores is limited. Its efficiency advantage was most pronounced in comparison to Workflow 2 (speech recognition), which had the longest documentation times.

## Discussion

### Interpretation of Findings

**Figure 7.**
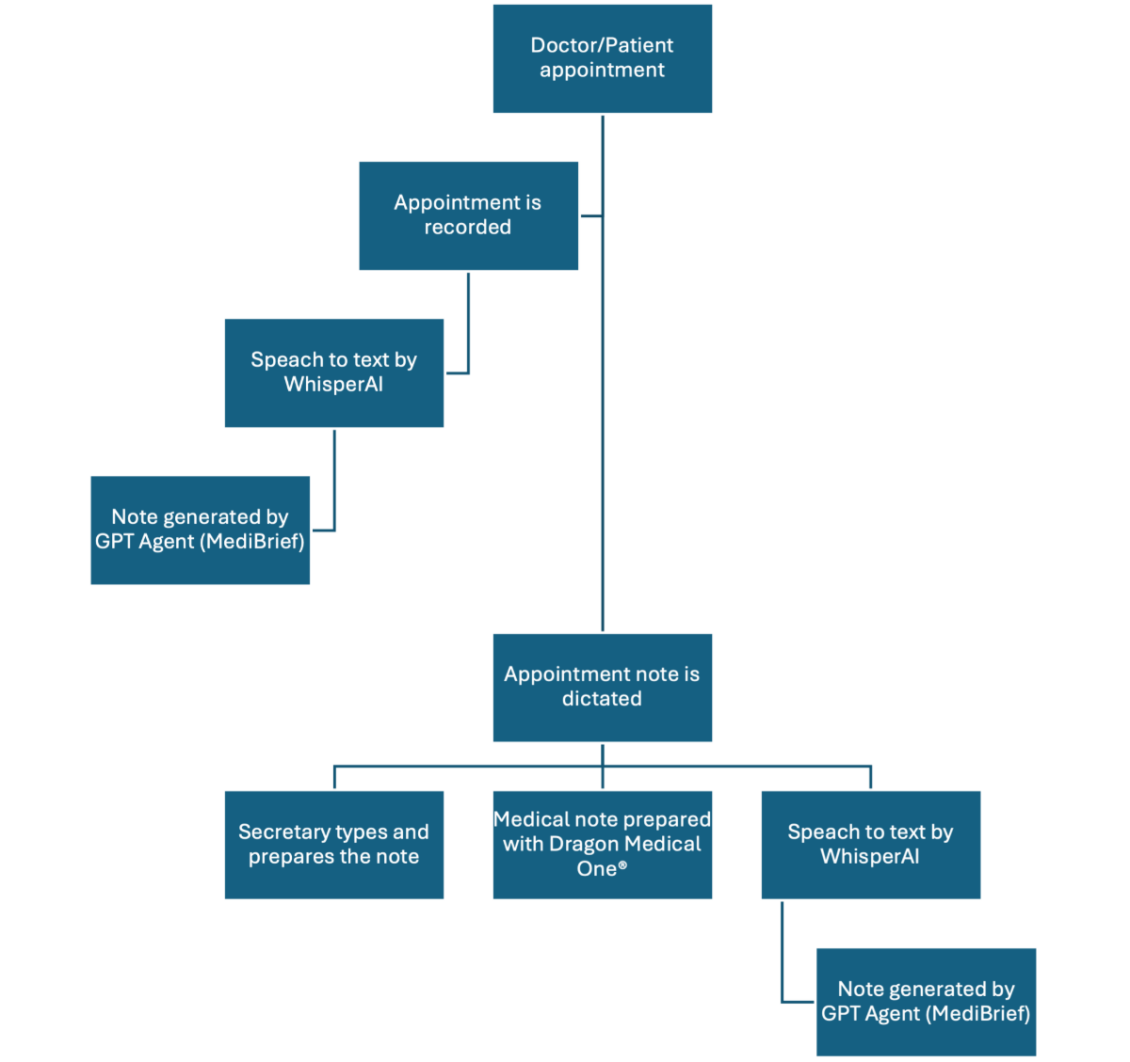
Workflows diagram. Schematic of the four documentation workflows, showing where speech-to-text and AI (artificial intelligence) formatting occur.

Our study confirmed that the ambient AI dictation workflow (Workflow 4) substantially reduced active documentation time for both physicians compared to the other methods, particularly versus the speech recognition workflow (Workflow 2). The time savings of Workflow 4 over traditional dictation (Workflow 1) did not reach statistical significance for the non-native physician (*P*=.08), indicating the study was underpowered to confirm this specific hypothesis. LLM evaluators assigned uniformly high PDQI-9 scores across all workflows. However, the negative Krippendorff’s α indicates systematic disagreement among the evaluators, meaning these absolute scores cannot be taken as reliable evidence of documentation quality. The quality assessment should be considered exploratory rather than definitive. It should be noted that scores for attributes such as “Accuracy” and “Thoroughness” reflect perceived plausibility rather than verified factual correctness, as the LLM evaluators had no access to the clinical events of the encounter (Figure 7).

### Clinical Importance of Differences

There is no established threshold for what constitutes a clinically important difference in PDQI scores in this context. In our series, observed differences were small and—critically—errors were easy to recognize and correct during brief review. A practical advantage of the AI workflows is that drafts are available immediately after the encounter, enabling same-moment correction, whereas secretarial pathways may introduce delays before physician review.

### Comparison With Prior Studies

Our findings on time efficiency align with prior research [20]. Independent benchmarking likewise reports near–real-time note generation yet nonzero error rates and sensitivity to multi-speaker/noisy conditions [7]. Quiroz et al [21] observed that AI-based digital scribe systems significantly reduced clinicians’ documentation time, supporting the idea that AI can automate much of the note-taking process. Similarly, Khalessi et al [22] reported a 51% decrease in consultation time when using an AI-powered assistant (OSLER), illustrating the streamlining of workflows when speech-to-text is integrated. These parallels in the literature reinforce that the efficiency gains we observed are achievable with AI assistance in documentation.

Our results regarding note quality are also consistent with other studies [20]. Bossen and Pine [23] described an AI “helper” that improved documentation accuracy and consistency when paired with human oversight [23]. In our study, physicians similarly reviewed and corrected AI-generated notes (Workflow 4), echoing the importance of human–AI collaboration to maintain reliability. Vogel et al [24] likewise found that speech recognition technology improved documentation speed and quality, which aligns with the performance of our Workflow 3 (Whisper+GPT) in achieving faster note completion than traditional dictation. Taken together, these studies underscore that integrating advanced speech-to-text and AI generation tools can enhance efficiency without compromising quality, in agreement with our findings.

Furthermore, the role of AI in multilingual health care environments (like our Swiss setting) remains underexplored. Recent work by Kalra and Seitzinger [25] suggests that AI can help bridge communication gaps and reduce errors in linguistically diverse settings. Our observation that the non-native speaker benefited most in terms of time savings supports this notion. It indicates that AI documentation tools may be especially valuable where language barriers would otherwise make documentation more cumbersome.

Overall, our results contribute to the growing body of evidence that AI-assisted documentation can improve efficiency without compromising quality. We also extend this literature by evaluating AI’s impact in a multilingual health care environment, demonstrating the potential for AI to alleviate documentation burdens in linguistically diverse settings.

### Practical Implications

The results of this study carry several practical implications. First, adopting AI-assisted documentation tools can substantially improve efficiency and reduce physicians’ administrative workload, which in turn may help lower the risk of physician burnout. By streamlining documentation, physicians can devote more time to direct patient care, potentially improving patient satisfaction and care quality. This benefit is especially pertinent in multilingual health care environments, where linguistic diversity complicates communication and documentation. In such settings, AI tools that proficiently handle multiple languages and dialects can bridge communication gaps and ensure more complete and accurate documentation across language barriers. Because performance differs across vendor implementations, institutions should validate candidate systems locally before rollout [7]. Our results also illustrate that not all IT-based documentation solutions reduce physicians’ workload: the speech-recognition workflow (Workflow 2) removes secretary time but *increases* physicians’ active editing time compared with traditional dictation to a secretary, effectively shifting rather than eliminating documentation work (Figure 5).

The design of our study—with simulated patients spanning multiple dialects and languages—also strengthens its real-world relevance. By mirroring the linguistic diversity that physicians face in Swiss health care, we were able to test AI documentation tools under realistic multilingual conditions. Notably, the AI systems maintained robust performance across this linguistic variability, indicating that such tools could be effective in actual diverse clinical environments. At the same time, our observations highlight that variations in patient language (different dialects or accents) may impact the efficiency and accuracy of speech recognition. Future research should investigate how specific dialects or accents affect AI performance and whether developing speech recognition models tailored to regional dialects can further enhance documentation outcomes.

Furthermore, the significant time savings observed, especially for the non-native German-speaking physician, suggest that AI-assisted documentation can level the playing field for clinicians working in non-native languages. This could lead to increased job satisfaction, reduced stress, and better retention of health care professionals in linguistically diverse settings.

### Implementation Considerations (Not Measured)

We did not collect data on costs, infrastructure dependencies, or training/onboarding effort; these domains will materially influence real-world value and should be quantified in implementation studies.

### Limitations

Despite our encouraging findings, several limitations must be considered.

### Use of Synthetic Data

Our study relied on synthetic (simulated) patient encounters rather than real patient interactions. Simulated patients were residents/interns familiar with the scenarios. This likely produced more structured, interruption-free speech than typical clinical visits and could favor transcription and summarization. This decision was driven by privacy concerns—especially for Workflow 4, which involved recording entire appointments in the cloud—but it means that the controlled simulation may not capture all the complexity and unpredictability of actual clinical settings. Real encounters often include overlapping speakers, disfluencies, background noise, and non-linear narratives; these conditions may degrade performance relative to what we observed here. As a result, the generalizability of our results to real-world practice needs careful interpretation.

### Generalizability Beyond Surgical Specialties

Our setting (Plastic & Hand Surgery) features relatively structured encounters. In more narrative-heavy domains (eg, internal medicine, neurology, psychiatry), speech characteristics and note structure differ, and AI advantages—and error profiles—may shift. Results should be extrapolated cautiously to those fields.

### Speech-to-Text Optimization

Workflow 2 (Dragon Medical One®) and our AI-assisted workflows (Whisper Large v3 in Workflows 3‐4) were *used without training/fine-tuning or site-specific optimization*. This likely *underestimates* the performance achievable with personalization (eg, specialty lexicons, microphone standardization) and domain-specific model tuning.

### Quality Assessment by AI

Our method of quality assessment has inherent limitations arising from the absence of independent clinical expert review. In routine practice, the ideal judge of documentation quality is the treating or referring physician with direct patient knowledge. In our study, we lacked such human clinical evaluators and instead used three different LLMs to score note quality. While using multiple AI reviewers helped minimize individual bias, it cannot replicate the nuanced clinical judgment of a physician. For the two attributes scored against Workflow 2 (“Free from Hallucination” and “Free from Bias”), the physician-approved note served as a pragmatic reference standard; however, residual speech-recognition errors that escaped physician correction could propagate as false ground truth, potentially penalizing other workflows for deviating from an error rather than introducing one. This design also creates circularity, as the reference standard is itself one of the comparators. For the remaining eight PDQI-9 attributes, the LLMs evaluated each note without any external reference, meaning scores for “Accuracy” and “Thoroughness” reflect perceived plausibility rather than verified factual correctness. Furthermore, the lack of variance in accuracy scores suggests a ceiling effect or lack of sensitivity in the LLM scoring tool. Future studies should incorporate independent clinical expert review to provide a true ground-truth assessment.

### Small Sample Size

The small sample size (two physicians and 12 simulated encounters) limits the diversity of clinical scenarios and clinician behaviors represented. Although a sample of twelve is adequate for pilot studies aimed at estimating variability, our results should be considered exploratory and not generalizable [26]. We were not powered for dialect/language subgroup analyses; actor-case assignments were not tracked to enable such stratification post hoc. Additionally, we did not systematically record actor-case assignments regarding dialects, which introduces an uncontrolled confounding variable. Critically, because only one physician represented each language group, the between-group factor (native vs non-native) is confounded with individual physician characteristics such as typing speed, system familiarity, and personal documentation style. Observed differences attributed to language background may therefore reflect individual traits rather than a true language effect, constituting a form of pseudoreplication. Future studies should include multiple physicians per language group to disentangle individual variability from language-related effects.

### Participant and Software Familiarity

The participating physicians’ familiarity with the cases and comfort with technology, as well a personal knowledge of “acting residents” may have positively influenced performance, and individual factors (like typing speed or prior experience with AI tools) could have impacted efficiency outcomes. Thus, our findings may not fully represent the wider physician population or more varied clinical environments. The timekeeper and participants were not blinded to workflows, which may introduce Hawthorne effects.

### Future Directions

To build on our findings, future studies should address the noted limitations. Real patient encounters—conducted with strict ethical oversight and data protection—are a priority for validating AI-assisted documentation in practice. In such trials, involving institutional review boards early and collaborating with data privacy officers will help ensure compliance with all regulations. It will also be important to include evaluations by independent clinicians (eg, referring physicians or external experts) to obtain clinically grounded assessments of note quality, rather than relying solely on AI or internal review.

Further research should also broaden the participant pool and case diversity. Studies that enroll more physicians across different specialties, experience levels, and linguistic backgrounds (and include a wider range of patient scenarios) will enhance the generalizability of results and reveal how AI documentation tools perform across various settings and user groups.

From a technological standpoint, exploring solutions that minimize data security risks is essential. On-premises AI systems or cloud services with robust compliance certifications could be tested to alleviate privacy concerns associated with cloud processing of sensitive patient data. Developing or fine-tuning AI models to run within a hospital’s secure IT infrastructure may allow institutions to harness AI benefits while maintaining full control over patient information.

Lastly, future investigations should examine the long-term, system-level effects of AI-assisted documentation. Key metrics could include patient throughput (eg, wait times or appointment lengths), physician satisfaction and well-being, and overall health care delivery efficiency. By tracking patient outcomes and workflow metrics over time, researchers can determine whether the immediate efficiency gains we observed translate into meaningful improvements in care and provider experience in the long run.

### Conclusion

In conclusion, AI-assisted documentation—particularly ambient AI dictation—demonstrated significant time savings without compromising documentation quality in a multilingual Swiss setting. These preliminary findings support further development and evaluation of AI scribes to alleviate documentation burden and improve equity for non-native speakers. Robust validation with larger samples, real patient encounters, human evaluators and secure data-processing frameworks is essential before routine deployment.

## Abbreviations

AI: artificial intelligence
EHR: electronic health record
GDPR: General Data Protection Regulation
LLM: large language model
PDQI-9: Physician Documentation Quality Instrument (9-item adaptation)
RME: relative marginal effect
WF1–WF4: documentation workflows 1‐4
